# Interaction of obesity polygenic score with lifestyle risk factors in an electronic health record biobank

**DOI:** 10.1186/s12916-021-02198-9

**Published:** 2022-01-12

**Authors:** Hassan S. Dashti, Nicole Miranda, Brian E. Cade, Tianyi Huang, Susan Redline, Elizabeth W. Karlson, Richa Saxena

**Affiliations:** 1 Center for Genomic Medicine, Massachusetts General Hospital and Harvard Medical School, Boston, MA USA; 2 Broad Institute, Cambridge, MA USA; 3 Department of Anesthesia, Critical Care and Pain Medicine, Massachusetts General Hospital and Harvard Medical School, Boston, MA USA; 4 Division of Sleep and Circadian Disorders, Department of Medicine, Brigham and Women’s Hospital, Boston, MA USA; 5 Division of Sleep Medicine, Harvard Medical School, Boston, MA USA; 6 Channing Division of Network Medicine, Brigham and Women’s Hospital and Harvard Medical School, Boston, MA USA; 7 Mass General Brigham Personalized Medicine, Mass General Brigham HealthCare, Boston, MA USA; 8 Department of Medicines, Brigham and Women’s Hospital and Beth Israel Deaconess Medical Center, Harvard Medical School, Boston, MA USA; 9 Division of Rheumatology, Inflammation and Immunity, Brigham and Women’s Hospital, Boston, MA USA

**Keywords:** BMI, Genetic risk, Obesity, Electronic health records, Phenome-wide association study, Gene-lifestyle interaction, Lifestyle, Obesogenic behaviors

## Abstract

**Background:**

Genetic and lifestyle factors have considerable effects on obesity and related diseases, yet their effects in a clinical cohort are unknown. This study in a patient biobank examined associations of a BMI polygenic risk score (PRS), and its interactions with lifestyle risk factors, with clinically measured BMI and clinical phenotypes.

**Methods:**

The Mass General Brigham (MGB) Biobank is a hospital-based cohort with electronic health record, genetic, and lifestyle data. A PRS for obesity was generated using 97 genetic variants for BMI. An obesity lifestyle risk index using survey responses to obesogenic lifestyle risk factors (alcohol, education, exercise, sleep, smoking, and shift work) was used to dichotomize the cohort into high and low obesogenic index based on the population median. Height and weight were measured at a clinical visit. Multivariable linear cross-sectional associations of the PRS with BMI and interactions with the obesity lifestyle risk index were conducted. In phenome-wide association analyses (PheWAS), similar logistic models were conducted for 675 disease outcomes derived from billing codes.

**Results:**

Thirty-three thousand five hundred eleven patients were analyzed (53.1% female; age 60.0 years; BMI 28.3 kg/m^2^), of which 17,040 completed the lifestyle survey (57.5% female; age: 60.2; BMI: 28.1 (6.2) kg/m^2^). Each standard deviation increment in the PRS was associated with 0.83 kg/m^2^ unit increase in BMI (95% confidence interval (CI) =0.76, 0.90). There was an interaction between the obesity PRS and obesity lifestyle risk index on BMI. The difference in BMI between those with a high and low obesogenic index was 3.18 kg/m^2^ in patients in the highest decile of PRS, whereas that difference was only 1.55 kg/m^2^ in patients in the lowest decile of PRS. In PheWAS, the obesity PRS was associated with 40 diseases spanning endocrine/metabolic, circulatory, and 8 other disease groups. No interactions were evident between the PRS and the index on disease outcomes.

**Conclusions:**

In this hospital-based clinical biobank, obesity risk conferred by common genetic variants was associated with elevated BMI and this risk was attenuated by a healthier patient lifestyle. Continued consideration of the role of lifestyle in the context of genetic predisposition in healthcare settings is necessary to quantify the extent to which modifiable lifestyle risk factors may moderate genetic predisposition and inform clinical action to achieve personalized medicine.

**Supplementary Information:**

The online version contains supplementary material available at 10.1186/s12916-021-02198-9.

## Background

Precision medicine aims to prevent, treat, and manage obesity and related diseases through targeted therapies [1]. Personalized approaches are expected to yield more effective therapies and efficient use of existing resources [1]. The initial drive toward precision medicine for obesity has been through quantifying disease risk based on genetic profiles and the simultaneous understanding of both genetic and lifestyle risk largely in healthy, population-based cohorts. However, for the adoption of personalized medicine into healthcare practice, examining the transferability of obesity genetic findings in a healthcare setting is essential to inform clinical action.

Both genetic and lifestyle factors have considerable effects on obesity and related diseases. The first genome-wide association study (GWAS) identified associations of the obesity-susceptibility locus, *FTO*, with a 0.39-kg/m^2^ unit increase in BMI per risk allele [2, 3]. Subsequently, 97, and more recently 751, single nucleotide polymorphisms (SNPs) significant in GWAS combined as a weighted polygenic risk score (PRS) explained 2.2% and ~ 6.0% of BMI variance, respectively [4, 5]. Genetic predisposition to obesity may be attenuated by adhering to a healthy lifestyle [6–9]. For example, physical activity, adequate sleep duration, and consumption of a healthy diet have been observed to diminish obesity genetic risk conferred by *FTO* and the 97 SNPs for BMI [10]. Furthermore, twin studies also support the role of an obesogenic environment on the phenotypic effects of obesity-related genes [11]. Based on these findings from general community settings of population-based cohorts, emphasizing a healthy lifestyle among genetically at-risk individuals may be clinically impactful, but this approach has not been tested in patient biobanks. Genetic susceptibility to obesity has also been related to other common metabolic and non-metabolic diseases, including type 2 diabetes and obstructive sleep apnea, illustrating pleiotropy and a potential mediating role of obesity as a risk factor [2, 12, 13]. Elucidating the relationship between the genetics of obesity and other diseases may help to prioritize diseases that will inevitably increase in prevalence because of the obesity epidemic.

Electronic health record (EHR) clinical biobanks offer the advantage of examining patients with a range of comorbid conditions and remain underutilized for obesity research [14]. EHR biobanks are rapidly growing because they enable quick patient enrollment, cost-effective research, and robust phenotype ascertainment at scale. Unique to the Mass General Brigham (MGB) Biobank is the inclusion of lifestyle surveys [15]. The bridging of clinical information to biological specimens and health surveys [16] offers a resource for the simultaneous consideration of obesity genetic factors and lifestyle risk factors. In addition, the implementation of EHR biobanks across academic medical systems provides patient cohorts enriched for disease phenotypes including those that have an overall low prevalence in traditional population-based cohorts [17]. Systematically examining the relationships between obesity genetics and hundreds of clinical phenotypes through phenome-wide scans can reveal links with obesity that have been previously unknown due to limited statistical power [18–21].

In the present study, we first examined the transferability of obesity genetic findings, including (1) associations with BMI and (2) interactions with lifestyle risk factors in a large patient biobank in aggregate and then separately in subgroups of patients with the lowest and highest comorbidity burden to determine potential heterogeneity of effects across patients. Then, we conducted a hypothesis-free phenome-wide scan to catalog obesity-disease links and to identify disease outcomes where a favorable lifestyle may attenuate risk conferred by a genetic predisposition to obesity.

## Methods

### Mass General Brigham Biobank

The Mass General Brigham (MGB) Biobank (formerly Partners Biobank) is a hospital-based cohort study from the MGB healthcare network in Boston, MA with electronic health record (EHR), genetic, and lifestyle data [15, 22, 23]. The MGB Biobank includes data obtained from patients in several community-based primary care facilities and specialty tertiary care centers in Boston, MA [15, 24]. The MGB network provides a wide range of healthcare services. Biobank patients are recruited from inpatient stays, emergency department settings, outpatient visits, and electronically through a secure online portal for patients. Recruitment and consent materials are fully translated in Spanish to promote patient inclusion. The systematic enrollment of patients across the MGB network and the active inclusion of patients from diverse backgrounds contribute to a Biobank reflective of the overall demographic of the population receiving care within the MGB network. Recruitment for the Biobank launched in 2009 and is ongoing through both in-person recruitment at participating clinics and electronically through the patient portal. The recruitment strategy has been described previously [15]. All recruited patients provided written consent upon enrollment. At the time of the analysis (03/2021), a total of 123,844 patients have consented. The present study protocol was approved by the MGB Institutional Review Board (#2009P002312, #2018P002276).

### Obesity polygenic risk score

A total of 43,446 patients have been genotyped with the Illumina Multi-Ethnic Genotyping Array and the Infinium Global Screening Array. The genetic data were harmonized, and quality controlled with a three-step protocol, including two stages of genetic variant removal and an intermediate stage of sample exclusion [25, 26]. The exclusion criteria for variants were (1) missing call rate ≥ 0.05, (2) minor allele frequency < 0.001, and (3) deviation from Hardy-Weinberg equilibrium (*P* < 1× 10^−6^). The exclusion criteria for samples were (1) sex discordances between the reported and genetically predicted sex, (2) missing call rates per sample ≥0.02, and (3) population structure showing more than four standard deviations within the distribution of the study population, according to the first four principal components (PCs). Phasing was performed with SHAPEIT2 [27] and imputations were performed with the Haplotype Reference Consortium Panel [28] using the Michigan Imputation Server [29]. Patient ancestry was determined using TRACE [30] with the Human Genome Diversity Project (HGDP) [31] as the reference panel. Principal component analysis outliers were determined by using a principal component analysis projection of the study samples onto the HGDP reference samples. To limit genetic heterogeneity in the present study, participants of non-European ancestry, which comprise only ~ 10% of the Biobank, were excluded from the analysis. To correct for population stratification, PCs were computed using TRACE [30] in genetically European participants. Among the participants with European ancestry, sample relatedness was inferred using KING [32], and subsequently, one sample from each related pair (kinship > 0.125) was randomly excluded.

A polygenic risk score (PRS) for obesity was generated for each patient based on 97 previously identified SNPs for BMI at the genome-wide significance level (*P* < 5× 10^− 8^) [4]. All SNPs had a minor allele frequency > 1% and an imputation quality (minimac *r*_sq_) ≥0.80 (Table S1). For each patient, the number of risk alleles weighted by the respective allelic effect sizes (β-coefficients) reported in the original GWAS meta-analysis [4] was summed. The score was subsequently scaled to allow interpretation of the effects as a per-1 BMI-increasing allele in the PRS (division by twice the sum of the β-coefficients and multiplication by twice the square of the SNP count representing the maximum number of risk alleles). The score was also standardized to have a mean of 0 and a standard deviation (SD) of 1 to allow comparison of the effects as per-1 SD with the obesity lifestyle risk index.

### Obesity lifestyle risk index

Following enrollment, Biobank participants were invited to complete an optional Health Information Questionnaire composed of lifestyle and family history questions (38.3% of Biobank participants responded [24]). For the present study, questions on 6 obesogenic lifestyle risk factors were considered to generate an obesity lifestyle risk index, including alcohol intake, education (as a proxy of socioeconomic status [33]), exercise, sleep habits, smoking, and shift work.

Specifically, *alcohol intake* was determined in response to the question, “During the past year how many alcoholic drinks (glass/bottle/can of beer; 4 oz glass of wine; drink or shot of liquor) did you usually drink in a typical week?”. Response options included none, or less than 1 per month, 1–3 per month, 1 per week, 2–4 per week, 5–6 per week, 1–2 per day, 3–4 per day, 5–6 per day, and more than 6 per day. *Education level* was reported in response to the question, “What is the highest grade in school that you finished?”. Response options included grade school (1–4 years), grade school (5–8 years), some high school (9–11 years), higher school diploma or GED (finished high school), some college, 2-year college or vocational school, 4-year college, and masters, doctoral or professional degree. *Exercise* was assessed with the question, “During the past year, what was your average time spent per week at each of the following recreational activities [bicycling; higher intensity exercise; jogging; lap swimming; lower intensity exercise; running; tennis, squash, or racquetball; walking or hiking (including to/from/for work)?”. Responses were aggregated to calculate total moderate to high-intensity exercise (excludes walking/hiking) in hour per week. *Sleep habits* were assessed with the questions, “In considering your longest sleep period, what time do you usually go to bed on weekdays or work or school days [or weekends or days off]?” and “In considering your longest sleep period, what time do you usually wake up on weekdays or work or school days [or weekends or days off]?”. Responses were in half-hour increments. Improbable reported bed and wake times were revised, consistent with previous analyses [24]. Sleep duration was then computed from bed and wake times with 5/7 weighting for weekdays and 2/7 for weekends. *Smoking* was assessed with the questions, “Have you smoked at least 100 cigarettes in your lifetime?”. Response options included, yes, currently smoke, yes, smoked in past, but quit, and no, have not smoked more than 100 cigarettes. Lastly, *shift work* was assessed with the question, “Which of the following best describes your usual work schedule?”. Response options included afternoon shift, night shift, irregular shift, rotating shift, split shift, and no shift (unemployed).

An obesity lifestyle risk index was constructed by aggregating exposure to the 6 obesogenic lifestyle risk factors: excessive or limited alcohol intake (more or less than 1 to 2 drinks per day [34, 35]), education level less than masters, doctoral or professional degree [36], physical inactivity (< 150 min of moderate- or high-intensity exercise per week [37]), inadequate sleep duration (< 8 h or ≥10 h per night [24]), former smoking (associated with higher odds of obesity compared to current and never smoking [38]), and night shift work [39]. To account for unequal effects of obesogenic lifestyle risk factors on obesity, the index was weighted to reflect the magnitude of the association of each trait with obesity, as previously conducted [40]. The weighting (effect sizes (β-coefficients)) of each trait was determined from an independent subset of MGB Biobank participants of self-reported European ancestry (*n* =30,045) that were otherwise excluded from the analysis because of the absence of genetic data (Additional files 1: Fig. S1, 2: Table S1). For each trait, the lowest risk category was assigned the reference group: moderate alcohol intake (1–2 drinks per day), highest education level (masters, doctoral or professional degree), recommended physical activity duration (≥150 min of moderate- or high-intensity exercise per week [37]), adequate sleep duration (≥8 and < 10 h per night), never smoking, and day shift work. Effect estimates were derived from a multivariable linear regression model for BMI including all 6 traits and adjusted for age and sex. Cross-trait correlations (*r*^2^) across the 6 traits ranged from − 0.22 to 0.15. For each participant, the respective effect estimates for all present obesogenic lifestyle trait were summed. The obesity lifestyle risk index was subsequently standardized to have a mean of 0 and a SD of 1 to allow interpretation of the effects as per-1 SD. A higher scaled index reflects more obesogenic behaviors.

### Body mass index, obesity status, and other disease outcomes

Body mass index (BMI) was calculated from participants’ measured height and weight by clinical staff during a clinical visit. For this analysis, the BMI closest to the date of Biobank enrollment was used.

Cases for obesity and other disease outcomes were determined from billing codes based on the International Classification of Diseases (ICD)-9/-10 diagnostic codes derived from all available EHR [15]. Both ICD-9 and ICD-10 were mapped to up to 1857 phenome-wide association study (PheWAS) codes (i.e., clinical phenotypes “phecodes”) based on clinical similarit y[41, 42]. For the obesity phecode, 278.1, the ICD-9 diagnostic codes were 278, 793.91, V85.3, V85.30, V85.31, V85.32, V85.33, V85.34, V85.54 and the ICD-10 diagnostic codes were E66.0, E66.09, E66.1, E66.8, E66.9, R93.9, Z68.30, Z68.31, Z68.32, Z68.33, Z68.34, Z68.54.

Same-day duplicated diagnoses and non-ICD-9/-10 codes were removed. To improve the positive predictive values for disease outcomes [43, 44], participants with at least 2 codes for any phecode were considered cases for that respective phenotype, whereas participants with no relevant code were considered controls. Relevant exclusionary diseases are curated lists of related conditions specific to each outcome (e.g., for Crohn’s disease, exclusionary diseases included ulcerative colitis and other related gastrointestinal complaints) aimed at generating robust control groups with limited case contamination to increase statistical power for finding associations [42, 45] and are listed in the PheWAS Catalog [44]. Participants with only one diagnostic code for a disease category or a code for any relevant exclusionary disease category were excluded from the analysis for that disease outcome. Thus, case-control sets for obesity phecode and every other disease outcome were unique.

### Statistical analysis

The analytical genetic sample included 33,511 unrelated patients of European ancestry with high-quality genetic data. First, we tested associations of the 97 SNPs for obesity, first separately then combined in the obesity PRS, with clinically measured BMI (primary outcome) in PLINK [46] using linear regression models and an additive genetic model adjusted for age, sex, genotyping array, and 5 PCs of ancestry. Following that, we tested for replication of the direction of effect of the 97 SNPs by performing a binomial test for the number of SNPs with the same direction of effect between the discovery study [4] and the present study (MGB Biobank) association results.

Among 17,040 adults with lifestyle information, we examined associations between the weighted obesity lifestyle risk index and BMI in linear regression models adjusted for age at survey completion and sex. We tested interactions between the obesity PRS and obesity lifestyle risk index on BMI by adding an interaction term between the PRS and the index and adding both the PRS and the index as covariates in addition to genotyping array and 5 PCs of ancestry in the multivariable linear regression models. To further examine the interaction, we dichotomized the obesity lifestyle risk index by the population median, and ran stratified association analyses of the obesity PRS with BMI in the high (more obesogenic behaviors) and low (less obesogenic behaviors) obesogenic subgroups.

In sensitivity analyses, we tested associations and interactions stratified by Charlson Comorbidity Index to examine the effect of comorbidity burden on study findings (low morbidity (healthiest): 10-year survival > 90.15%; high morbidity (sickest): 10-year survival= 0.009%). The Charlson Comorbidity Index, derived from EHR data, is a validated index that combines the presence and severity of comorbidities with age to predict the 10-year survival probability [47]. In addition, we tested associations between the obesity PRS and the obesity lifestyle risk index and its individual lifestyle factors; furthermore, we examined separate interactions with individual SNPs (97 SNPs) and individual lifestyle factors (6 factors) on BMI. Interactions with individual SNPs and individual lifestyle factors were considered significant at Bonferroni *P* value cut-offs accounting for the total number of interaction tests.

Next, we conducted a PheWAS for the obesity PRS with 675 other diseases using the PheWAS R package [45]. In aggregate, the analyzed patients had a total of 25,184,047 ICD-9 and ICD-10 diagnostic codes corresponding to 1,650,288 instances of phecodes (*n* =8349 for obesity phecode) with at least 2 distinct diagnostic codes. Only diseases with at least 1% case prevalence (i.e., *n* cases ≥ 335) were considered. We tested associations between the obesity PRS and each of 675 diseases using logistic regression with adjustments for age, sex, genotyping array, and 5 PCs of ancestry, then further adjusted for BMI. Associations were considered significant at Bonferroni *P* value cut-offs accounting for the total number of tested diseases (i.e., cross-sectional analysis *P* value = 0.05/675 tested diseases with at least 1% case prevalence =1.49 × 10^−4^). For significant PheWAS findings, we conducted association tests comparing the highest (Q10) to lowest (Q1 - reference) decile of the obesity PRS to demonstrate effect differences in patients in the extreme tails of the risk distribution. We then systematically conducted interaction tests between the obesity PRS and obesity lifestyle risk index for all disease outcomes significantly associated with the obesity PRS in the PheWAS by further adding an interaction term between the PRS and the index and adding both the PRS and the index as covariates. Interactions were considered significant at the Bonferroni threshold of *P* < 0.00125 accounting for 40 disease outcomes (the number of significant associations from the PheWAS). In sensitivity analyses, we stratified PheWAS by Charlson Comorbidity Index to examine the effect of morbidity on interaction findings. To partly account for potential changes in lifestyle attributed to disease onset, in additional sensitivity interaction analyses, we only included new diagnoses made 1 year and later after Biobank enrollment.

All analyses were conducted using R (version 4.0.3; The R Foundation for Statistical Computing, Vienna, Austria).

## Results

**Fig. 1:**
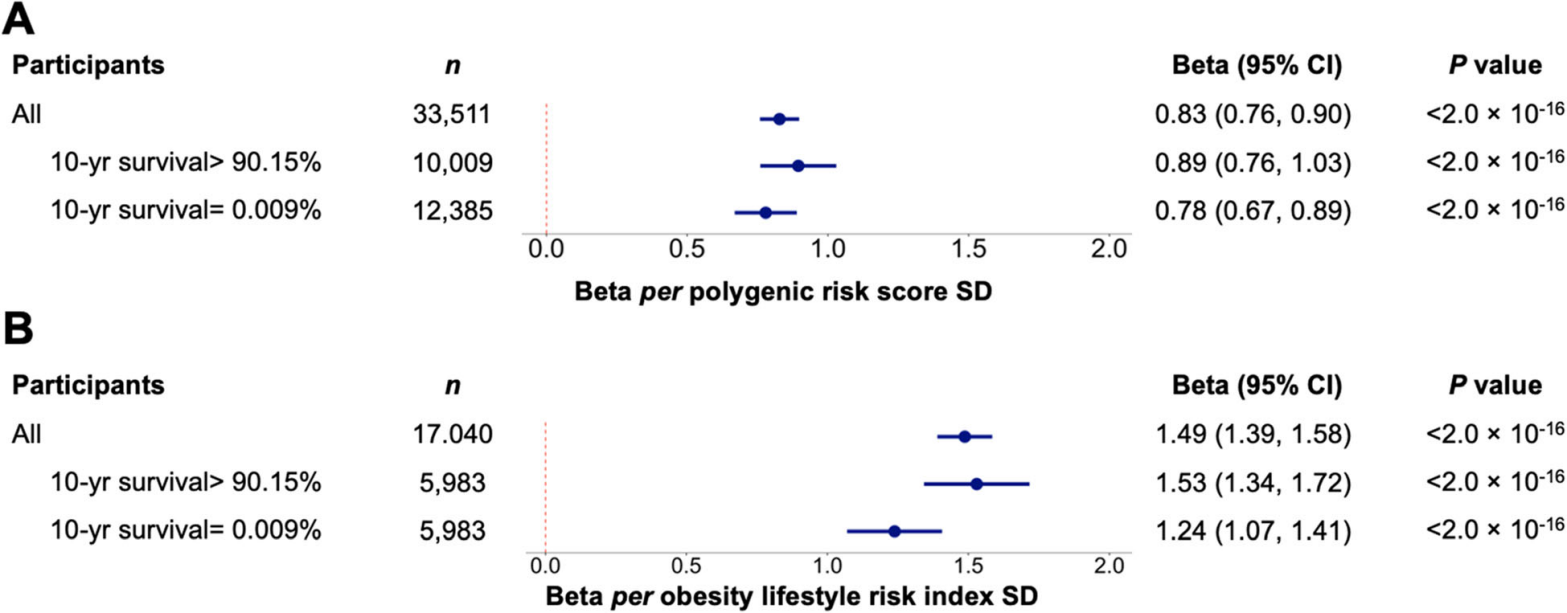
Associations of obesity genetic risk and obesity lifestyle risk index with clinically measured BMI with effect modification by comorbidity in the Mass General Brigham Biobank. **A** Associations of the obesity PRS with clinically measured BMI in all 33,511 patients and associations stratified by lowest and highest morbidity based on the Charlson Comorbidity Index (10-yr survival probability). Effect estimates are derived from a multivariable linear regression model for BMI adjusted for age, sex, genotyping array, and 5 PCs of ancestry per SD of the PRS. **B** Association of the obesity lifestyle risk index with clinically measured BMI in all 17,040 patients and associations stratified by lowest and highest morbidity. Effect estimates are derived from a multivariable linear regression model for BMI adjusted for age and sex per SD of the obesity lifestyle risk index. Abbreviations: polygenic risk score (PRS), principal components (PCs), standard deviation (SD)

A total of 33,511 adult patients of European ancestry from the MGB Biobank were included in the genetic analyses (Additional files 1: Fig. S1, 2: Table S1). Mean age was 60.0 years (SD =16.9), 53.1% were female, and mean BMI was 28.3 kg/m^2^ (SD =6.3). The median (range) for the number of BMI-increasing alleles was 90 (64, 117). Of the 97 BMI loci, the *FTO* locus had the strongest association with BMI (0.58 kg/m^2^ per effect allele). In total, 91 signals showed a direction of association concordant with the discovery GWAS (exact binomial test *P* = 6.2 × 10^−21^) (Additional files 1: Fig. S2A, 2: Table S2). The obesity PRS accounted for 2.9% of the variance in BMI. On average, each SD increment in the PRS was associated with 0.83 kg/m^2^ unit increase in BMI (95% confidence interval (CI) = 0.76, 0.90), and associations were observed among patients with the lowest and highest morbidity based on the Charlson Comorbidity Index (Fig. 1A). The average effect per BMI-increasing allele was 0.13 kg/m^2^ (95% CI =0.12, 0.14), and patients in the highest decile of the score had an average 2.87 kg/m^2^ higher BMI than patients in the lowest decile (Additional file 1: Fig. S2).

Of the genetic sample, 17,040 participants (57.5% female; mean (SD) age: 60.2 (16.4) years, SD =16.4; BMI: 28.1 (6.2) kg/m^2^) completed the lifestyle survey (Additional file 2: Table S1). The weighted obesity lifestyle risk index composed of 6 obesogenic lifestyle risk factors was associated with BMI (Additional file 1: Fig. S3) and accounted for 6.6% of the variance in BMI. On average, each SD increment in the index was associated with 1.49 kg/m^2^ unit increase in BMI (95% CI =1.39, 1.58), and patients in the highest decile of the score had an average 4.53 kg/m^2^ higher BMI than patients in the lowest decile (Fig. 1B). Associations were observed among patients with the lowest morbidity (1.53 kg/m^2^ per SD (95% CI =1.34, 1.72)) and the highest morbidity (1.24 kg/m^2^ per SD (95% CI =1.07, 1.41)). The obesity PRS was not associated with the obesity lifestyle risk index or any individual obesogenic lifestyle trait (all *P* > 0.05) (Additional file 2: Table S3).

**Fig. 2:**
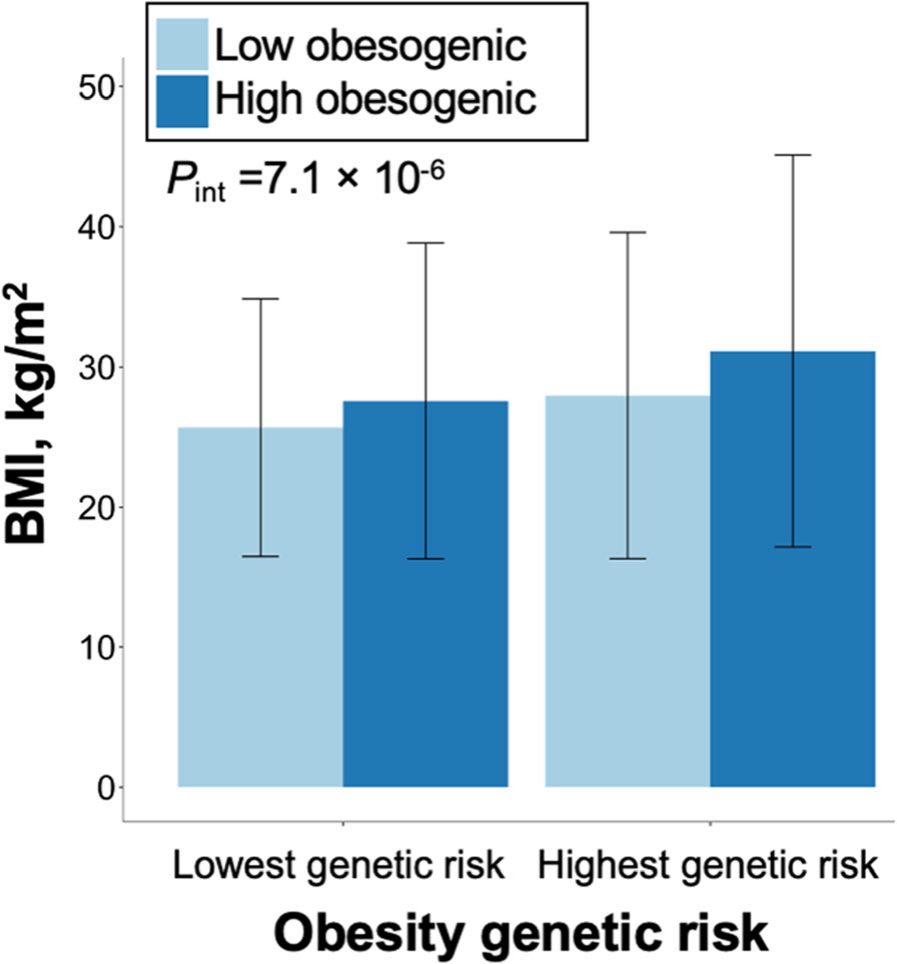
Average clinically measured BMI by lowest and highest decile of obesity genetic risk and by obesity lifestyle risk index in an electronic health record biobank (*n* =17,040). *P*_int_ value is for the interaction term between the PRS and the obesity lifestyle risk index (both continuous) on BMI in a multivariable linear regression model adjusted for age, sex, genotyping array, and 5 PCs of ancestry adding both the PRS and the index as covariates. The obesity lifestyle risk index was standardized to have a mean of 0 and a standard deviation of 1 then dichotomized by the median and presented as low (less obesogenic behaviors) and high (more obesogenic behaviors). Abbreviations: polygenic risk score (PRS), principal components (PCs)

**Fig. 3:**
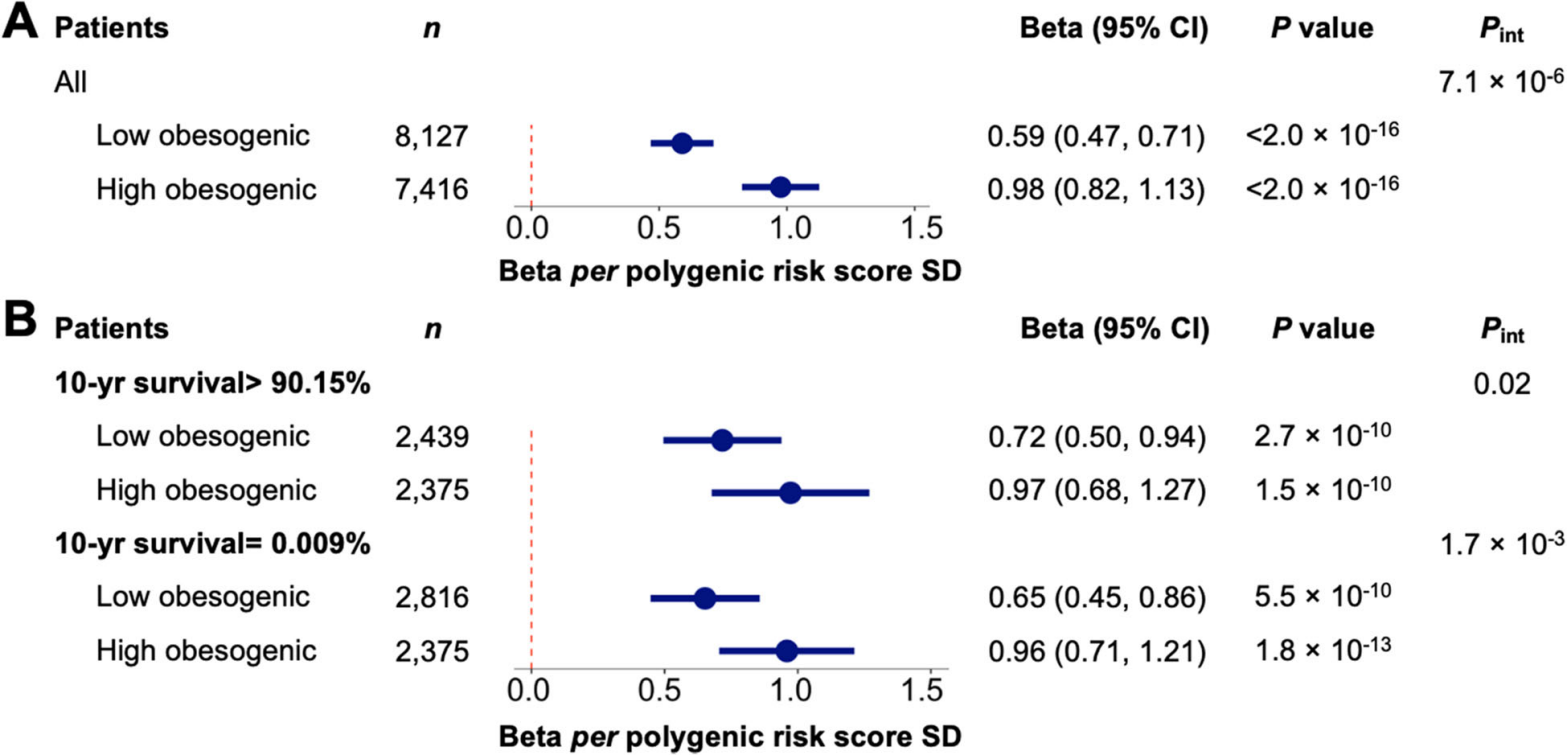
Interaction between obesity genetic risk and obesity lifestyle risk index on clinically measured BMI in an electronic health record biobank (*n* =17,040). **A** Interactions and associations of the obesity PRS with clinically measured BMI stratified by low and high obesity lifestyle risk index (low vs. high obesogenic behaviors). Effect estimates (Beta) are derived from a multivariable linear regression model for BMI adjusted for age, sex, genotyping array, and 5 PCs of ancestry per SD of the polygenic risk score. *P*_int_ value is for the interaction term between the PRS and the obesity lifestyle risk index (both continuous) on BMI in the multivariable linear regression model with the PRS and the index added as covariates. **B** Interaction and associations stratified by lowest and highest morbidity based on the Charlson Comorbidity Index (10-yr survival probability). Effect estimates are derived from a multivariable linear regression model for BMI adjusted for age, sex, genotyping array, and 5 PCs of ancestry per SD of the PRS. *P*_int_ value is for the interaction term between the PRS and the obesity lifestyle risk index (both continuous) on BMI in a multivariable linear regression model with the PRS and the index added as covariates. Abbreviations: confidence interval (CI), polygenic risk score (PRS), principal components (PCs), standard deviation (SD)

There was an interaction between the obesity PRS and obesity lifestyle risk index on BMI (*P*_int_ = 7.1 × 10^−6^). The association of a favorable lifestyle with lower BMI was larger in patients with a high genetic predisposition to obesity than in patients with a low genetic predisposition. Specifically, among patients with the highest obesity genetic risk (highest decile), the difference in BMI between those with a high and low obesity lifestyle risk index was 3.18 kg/m^2^, whereas among patients with the lowest obesity genetic risk (lowest decile), the difference in BMI between those with a high and low obesity lifestyle risk index was only 1.55 kg/m^2^ (Fig. 2). Presented differently, among patients with a high index (more obesogenic behaviors), the obesity PRS effect per SD increment was 0.98 kg/m^2^ (95% confidence interval (CI) =0.82, 1.13) kg/m^2^, whereas among patients with a low index (less obesogenic behaviors), the obesity PRS effect per SD increment was only 0.59 kg/m^2^ (95% CI =0.47, 0.71) kg/m^2^ (Fig. 3A). The interaction between the PRS and obesity lifestyle risk index on BMI was observed among patients with the lowest (*P*_int_ = 0.02) and the highest (*P*_int_ = 1.7 × 10^−3^) morbidity (Fig. 3B). In sensitivity analyses, among the BMI loci, interactions were strongest for *FTO* (*P*_int_ =2.4 × 10^−4^) and *CADM2* (*P*_int_ =1.4 × 10^−3^), and among the obesogenic lifestyle risk factors, individual interactions were evident for exercise (*P*_int_ =1.8 × 10^−4^), alcohol intake (*P*_int_ =3.7 × 10^−3^), and education (*P*_int_ =0.01) (Additional file 1: Fig. S4, S5).

**Fig. 4:**
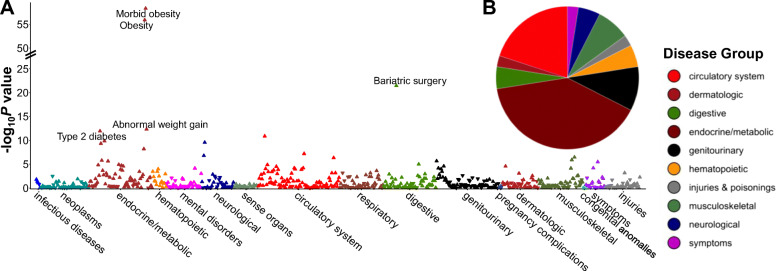
Phenome-wide association results for the obesity PRS (*n* =33,511). **A** Manhattan plot showing phenome-wide associations between the obesity PRS and 675 disease outcomes grouped by their broad disease groups on the *x*-axis and the -log_10_*P* value of the association on the *y*-axis. The horizontal red line represents the Bonferroni corrected *P* value cut-off (*P* value =1.49 × 10^−4^). Each disease outcome is represented by either an upward or downward triangle indicating a positive or negative association, respectively. **B** Pie chart summarizing distribution of significant PheWAS findings across disease groups. Abbreviations: phenome-wide association study (PheWAS), polygenic risk score (PRS)

**Fig. 5:**
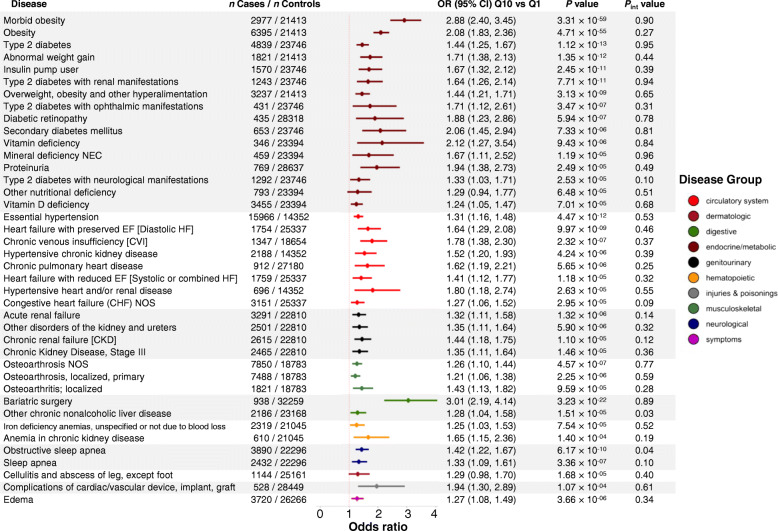
Phenome-wide associations between obesity PRS and disease outcomes and interactions between obesity PRS and obesity lifestyle risk index on disease outcomes. Disease outcomes were limited to 40 significant findings from obesity PRS PheWAS. Disease outcomes are color-coded by their corresponding disease groups as described in the shared legend. PheWAS association models were adjusted for age, sex, genotyping array, and 5 PCs of ancestry. PheWAS association results are presented as OR (95%) and corresponding *P* value comparing highest (Q10) to lowest (Q1 - reference) decile of the obesity PRS. In interaction analyses, an interaction term between the PRS and the index was added and both the PRS and the index were added as covariates. *P*_int_ value are *P* values for the interaction term between the continuous PRS and obesity lifestyle risk index. Interactions were considered significant at *P*_int_ < 0.00125 accounting for 40 tests. Abbreviations: odds ratio (OR), phenome-wide association study (PheWAS), polygenic risk score (PRS), quartile (Q)

PheWAS results for the obesity PRS and 675 disease outcomes including 33,511 patients (Additional file 2: Table S1) are presented in Additional file 2: Table S4. Associations were evident for 40 disease outcomes spanning endocrine/metabolic (40.0% of total findings), circulatory system (20.0%), and 8 other disease groups (Fig. 4). The 5 most significant associations were for morbid obesity (PRS Q10 to Q1 odds ratio (OR) (95% CI): 2.88 (2.40, 3.45)), obesity (2.08 (1.83, 2.36)), bariatric surgery (3.01 (2.19, 4.14)), type 2 diabetes (1.44 (1.25, 1.67)), and abnormal weight gain (1.71 (1.38, 2.13)) (Fig. 5). On average, each SD increment in the PRS was associated with 1.26 (95% CI =1.22, 1.29) higher odds of obesity diagnosis and was evident among patients with the lowest and highest morbidity. Associations for some signals were attenuated upon adjusting for BMI, suggesting disease risk is likely to be mediated through obesity (Additional file 2: Table S4 ). No significant interactions were observed between the obesity PRS and obesity lifestyle risk index on obesity diagnosis (phecode) (*P*_int_ =0.27) or any other disease outcome identified in PheWAS (all *P*_int_ > 0.03) (Fig. 5, Additional file 2: Table S5).

## Discussion

In an analysis of adult patients in a clinical biobank, we observed (1) that an obesity PRS was robustly associated with clinically measured BMI; (2) an interaction between an obesity PRS and an obesity lifestyle risk index, such that among patients with a higher obesity genetic risk, an obesogenic lifestyle exacerbated the genetic risk, regardless of patient morbidity; (3) in PheWAS, that an obesity PRS was associated with novel and known diseases spanning multiple categories; and (4) that an obesogenic lifestyle did not modify the associations between an obesity PRS and disease outcomes derived from billing codes. Overall, the results of this study emphasize the beneficial effect of reducing obesogenic behaviors particularly among patients with high obesity genetic risk, demonstrate the pleiotropic nature of obesity genetics suggesting novel mechanistic links between obesity and other diseases, and highlight limitations of leveraging clinical biobanks in advancing precision medicine research.

First, we show strong transferability of genetic findings for obesity from generally healthy population-based cohorts to a patient-centered clinical EHR biobank. The BMI SNPs had effects largely concordant with those identified in population-based cohorts and the obesity PRS explained variance in BMI comparable with that reported in population-based cohorts (EHR = 2.9%; population-based cohort = 2.1%) [4]. Among the 97 variants, the *FTO* locus showed the most prominent effect on BMI [3]. The consistency of genetic effects was observed despite fundamental cohort differences in BMI ascertainment (height and weight from clinical visits by clinical staff vs. height and weight from controlled research visits typically by trained research staff according to standard guidelines) and clinical factors (hospital-based vs. healthy population-based cohort). The transferability of genetic findings in EHR biobanks supports the continued use of rapidly growing EHR biobanks in advancing obesity research.

Next, we demonstrate interactions between an obesity PRS and an obesity lifestyle risk index in an EHR biobank, extending findings from healthy adults to patients with a range of comorbidities [48, 49]. Gene-lifestyle interactions have primarily been conducted in healthy population-based cohorts [9, 48, 50], which are susceptible to selection bias [51]. Reported interactions include those between *FTO* and physical activity in adults where physical activity attenuated the effects of the *FTO* effect allele on obesity from an odds ratio of 1.30 to 1.22 per effect allele [48]. Similarly, the difference in BMI between adults who regularly consumed fried foods compared to those that didn’t was 1.0 kg/m^2^ for women and 0.7 kg/m^2^ men with high obesity genetic risk, but only 0.5 kg/m^2^ for women and 0.4 kg/m^2^ for men with low genetic risk [50]. In addition, adults with low quality diets had a 1.14 kg/m^2^ higher BMI per 10-unit increment in a BMI PRS, compared to only 0.84 kg/m^2^ higher BMI among adults with high quality diets [8]. In the present analysis, we found that among patients with a high index (more obesogenic behaviors), obesity genetics conferred a larger effect on BMI compared to patients with a low index (less obesogenic behaviors). Conversely, a more favorable lifestyle was associated with an attenuated genetic risk for elevated BMI conferred by the obesity PRS. The magnitude of the interaction effect reported in the present study is larger to what has been reported previously possibly due to the inclusion of additional common variants for obesity, aggregation of multiple lifestyle risk factors, or consideration of patients [8, 50]. Worth noting is that the obesity lifestyle risk index explained a greater proportion of variance in BMI than the obesity PRS (6.6% vs. 2.9%, respectively), highlighting the importance of routinely evaluating and monitoring lifestyle in healthcare settings. As the obesity PRS was not associated with individual components of the index, high obesity genetic risk does not predispose to the obesogenic behaviors included in the analysis but possibly to other lifestyle factors not considered such as diet. The gene-lifestyle interaction was robust in patients with the lowest and highest Charlson Comorbidity Index indicating that targeting obesogenic behaviors may produce favorable effects regardless of comorbidity burden. Overall, these findings add to the growing literature indicating that genetic predispositions to obesity are not deterministic, but may be modified by lifestyle [6–9]. Thus, genetic data could be leveraged in a healthcare setting to prioritize healthy lifestyle strategies in patients at greatest risk for obesity. As interactions were evident with multiple obesogenic behaviors independently, recommending moderate improvements to any, or all, obesogenic behaviors may be beneficial.

Through the application of PheWAS for the obesity PRS, we provide an atlas of disease outcomes associated with obesity genetic risk. Overall, we identified 40 signals, highlighting the pleiotropic nature of obesity genetics [52, 53]. Associations were consistently positive, suggesting that obesity genetic risk likely increases risk for other diseases; however, causality cannot be inferred from the present analysis. The findings included known associations with type 2 diabetes and sleep apnea [54, 55]. We also identified novel associations with disease subphenotypes and other less prevalent diseases [56, 57]. For example, we observed associations between the PRS with both heart failure with reduced ejection fraction and heart failure with preserved ejection fraction. In addition, we identified novel associations with nutritional deficiencies, including vitamin D, iron, and more commonly, vitamins and minerals. These associations suggest that higher BMI is associated with decreased bioavailability of circulating micronutrients, specifically vitamin D and iron [58–60]. So far, Mendelian randomization analyses support a link between higher BMI and lower vitamin D status; causal links with other micronutrients may exist [58]. These findings emphasize the importance of examining the nutritional status of obese individuals, specifically among those with bariatric surgery, and addressing possible deficiencies through healthy food choices or supplementation despite likely excessive dietary intake [61]. The future application of phenome-wide scans in clinical biobanks may continue to generate novel hypotheses and advancing translational research.

In interaction analyses using billing codes, we did not identify diagnoses where targeting obesogenic behaviors may attenuate disease risk conferred by obesity genetic variants. There was no detectable interaction for obesity diagnosis phecodes despite robust interactions between the PRS and obesity lifestyle risk index for BMI. The absence of an interaction for obesity diagnosis may suggest that the interaction for BMI may be statistically significant but clinically modest [62]. Alternatively, the lack of interaction may highlight general limitations in leveraging administrative data for research, including inaccurate and incomplete patient diagnoses contributing to case misclassification, particularly for obesity [63]. It is known that limiting obesogenic behaviors, including physical inactivity, inadequate sleep, and excessive alcohol consumption, reduce risk of cancer, diabetes, and cardiovascular diseases [52, 53, 64]. Thus, algorithms combining diagnosis and procedural codes along with other clinical values may lead to more precise phenotypic ascertainment.

Several additional limitations should be considered. The study was restricted to participants of European ancestry to limit genetic heterogeneity; future efforts in racially and ethnically diverse populations are necessary to allow the generalizability of findings and promote health equity. The PRS included in the analysis was limited to 97 genetic variants for BMI previously shown to interact with lifestyle, however, other interactions may exist for a PRS comprised of additional genetic variants identified in more recent GWAS [5] or a genome-wide PRS [65]. The optional lifestyle survey responders were generally more likely to be women and to have a lower 10-year survival probability than non-responders, and therefore selection bias may still exist. In addition, the survey was only administered once at enrollment, and therefore the stability of these behaviors over time is unknown. Also, the survey did not account for all known obesogenic behaviors, including diet, which has been shown to interact with obesity genetic risk [8, 50], and did not include data on other potentially relevant covariates, such as income. The obesogenic lifestyle risk index was based on crude lifestyle phenotyping from self-reported data and weighted according to the associations of lifestyle behaviors with BMI, which may not be generalized to other disease-specific lifestyle risk scores. The most appropriate method for developing global lifestyle risk indices and assessing their interaction with genetics has yet to be determined. While our phenome-wide scan is 50% larger than previous efforts [52], there remain several rare diseases that were excluded because of inadequate number of cases and likely limited power. Finally, all analyses were cross-sectional and should be interpreted cautiously given that participants were patients who may have changed their behaviors upon diagnosis and given that our findings do not indicate that changing behaviors according to our obesity lifestyle risk index resulted in improved disease outcomes.

## Conclusions

By considering the potential interplay between gene and lifestyle choices in a clinical biobank, we provide evidence in patients that support both the role of genetic susceptibility and lifestyle in obesity risk. Moreover, we show evidence of a significant interaction between genetic and lifestyle risk factors for BMI, suggesting that emphasizing modifiable lifestyle behaviors to patients may attenuate risk conferred by common genetic variants associated with obesity. These findings highlight non-pharmacological behavior change therapies as potential treatments for a complex disease in a clinical setting. Through phenome-wide scans, we also provide evidence linking genetic susceptibility to elevated BMI with diseases spanning multiple categories. Continued evaluation of the role of lifestyle in the context of genetic predisposition is warranted to support the full potential of personalized medicine.

## Supplementary Information

**Additional file 1: Figures S1-S5.** **Fig S1.** – [Flow chart, patients analyzed in the MGB Biobank]. **Fig S2.** – [Associations of BMI SNPs and obesity PRS with BMI]. **Fig S3.** – [Associations of obesogenic lifestyle risk factors and obesity lifestyle risk index with BMI]. **Fig S4.** – [Interactions between 97 BMI SNPs and obesity lifestyle risk index on BMI]. **Fig S5.** – [Interactions between obesity PRS and obesogenic lifestyle risk factors on BMI and associations between obesity PRS per SD and BMI by lifestyle risk factor risk].

**Additional file 2: Tables S1-S5.** **Table S1.** – [Characteristics of participants in MGB Biobank]. **Table S2.** – [97 BMI SNP associations with BMI (in kg/m2) in MGB Biobank]. **Table S3.** – [Associations of obesity PRS per SD with obesity lifestyle risk index and risk factors]. **Table S4.** – [Cross-sectional PheWAS results for obesity PRS in all patients in the MGB Biobank]. **Table S5.** – [phenome-wide interactions between obesity PRS and obesity lifestyle risk index on disease].

## Data Availability

Data are available from the Mass General Brigham Human Research Office/Institutional Review Board at Mass General Brigham (contact located at https://www.partners.org/Medical-Research/Support-Offices/Human-Research-Committee-IRB/Default.aspx) for researchers who meet the criteria for access to confidential data.

## Abbreviations

BMI: Body mass index
EHR: Electronic health record
ICD: International classification of diseases
MGB: Mass General Brigham
PheWAS: Phenome-wide association study
PRS: Polygenic risk score
SNP: Single nucleotide polymorphism
