# Supplementary material for: Interaction of obesity polygenic score with lifestyle risk factors in an electronic health record biobank
Source: BMC Med. 2022 Jan 12;20:5. doi: 10.1186/s12916-021-02198-9 (PMC8753909; doi:10.1186/s12916-021-02198-9)

**Fig. S1 Flow chart of patients analyzed in the Mass General Brigham Biobank.**


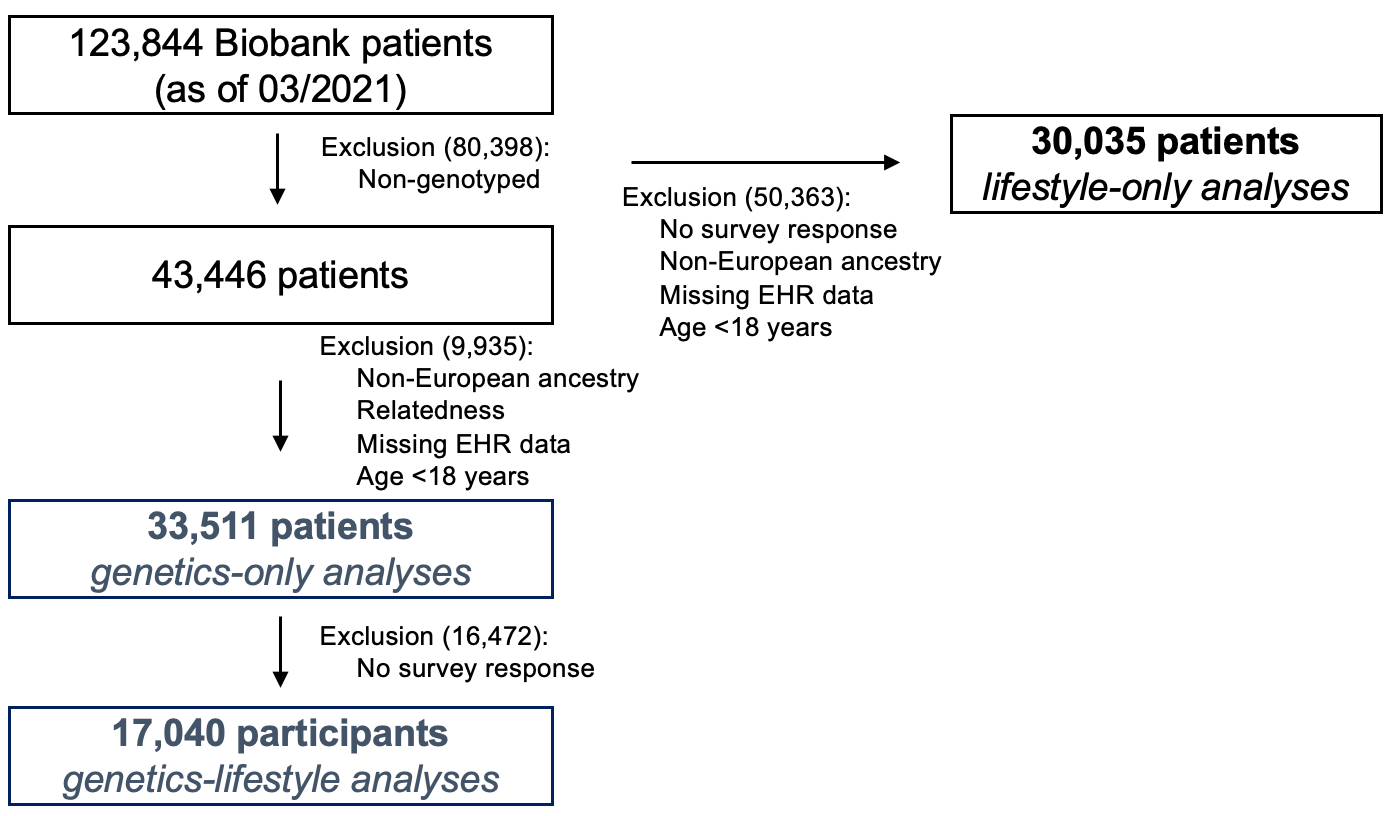


**Fig. S2 Associations of 97 BMI SNPs and obesity polygenic risk score with clinically measured BMI in kg/m^2^ (*n* =33,511).** A) Associations of the 97 BMI SNPs are from additive genetic models adjusted for age, sex, genotyping array, and 5 PCs of ancestry in kg/m^2^. The solid horizontal blue line represents the Bonferroni corrected *P* value cut-off (*P* value =5.15x10^-4^). The dashed vertical red line indicates effect estimate of 0. B) Distribution (*n*) of the weighted polygenic risk score and average measured BMI per each 10-point interval in the polygenic risk score.

**
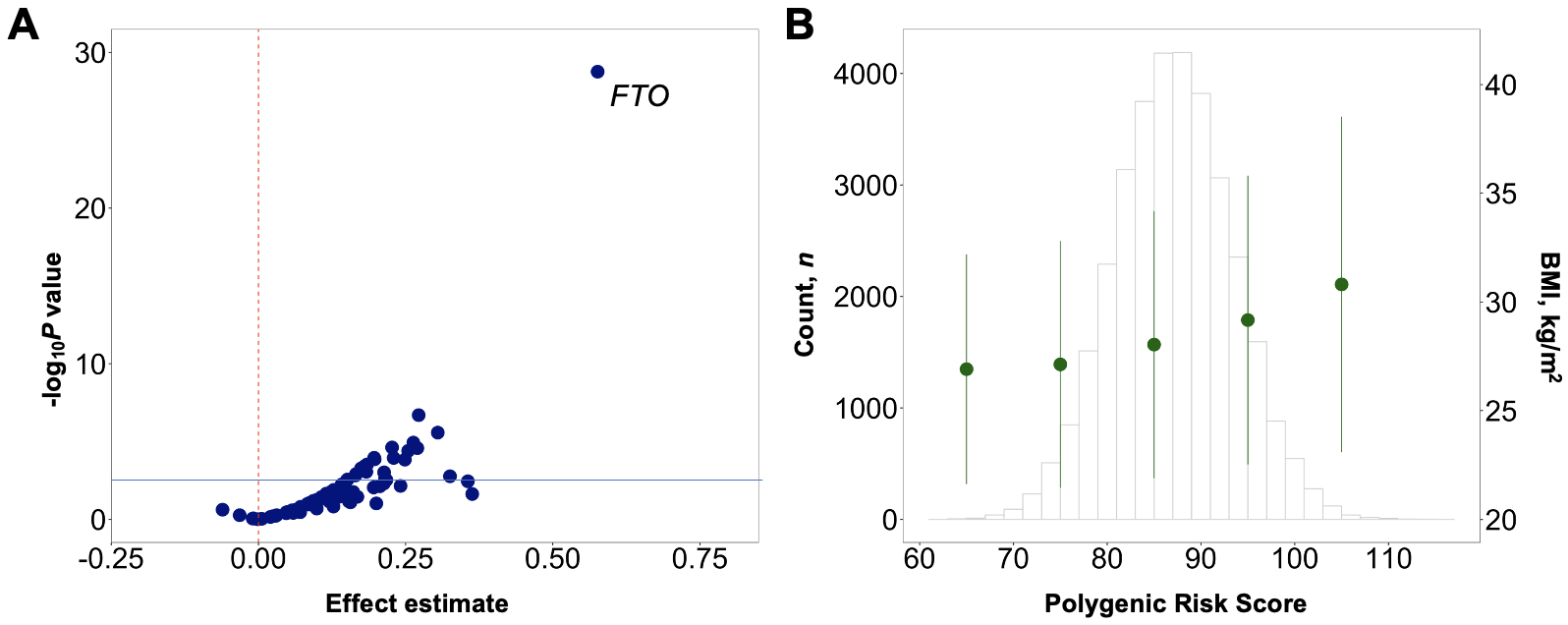
**

**Fig. S3 Associations of obesogenic lifestyle risk factors and obesity lifestyle risk index with clinically measured BMI in an electronic health record biobank (*n* =17,040).** A) Associations of obesogenic lifestyle risk factors with clinically measured BMI in kg/m^2^. Association results are from linear regression models adjusted for age at survey completion and sex. B) Distribution of the obesity lifestyle risk index and average measured BMI per each 1-standard deviation interval in the index. Abbreviations: confidence interval (CI), standard deviation (SD).


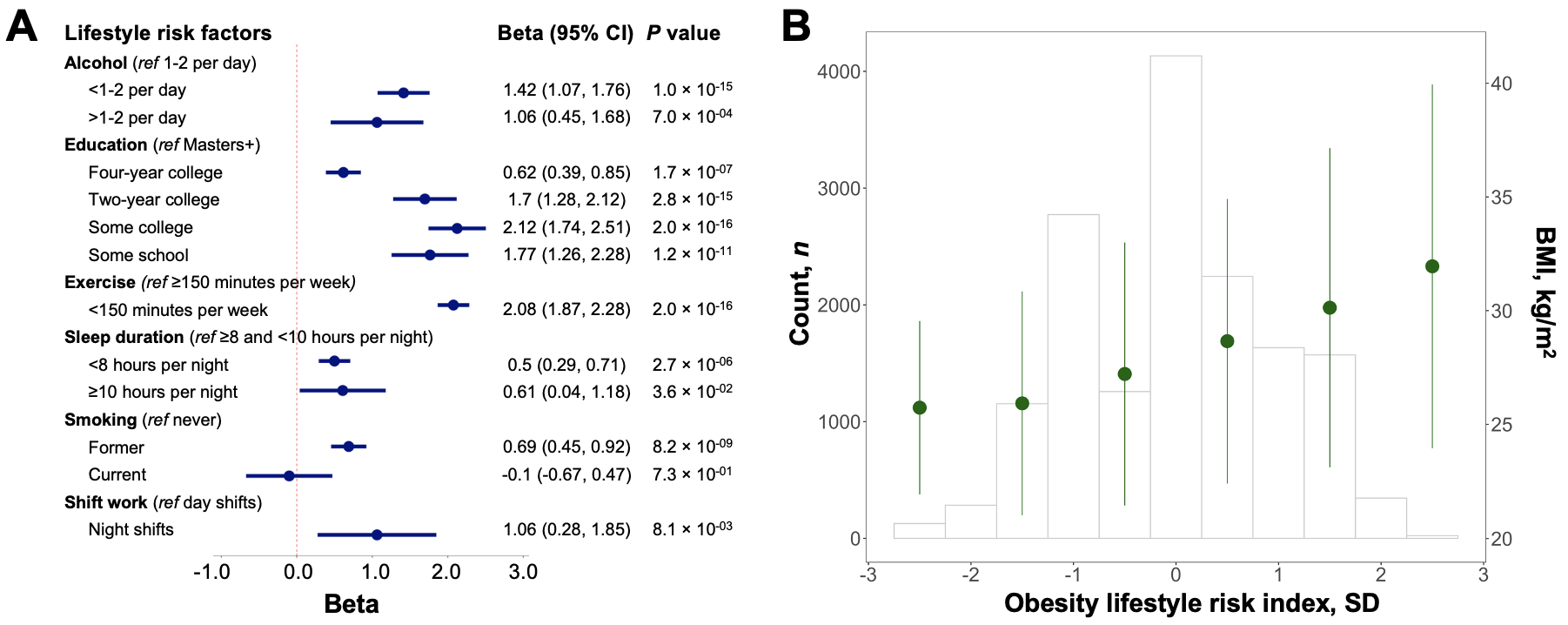


**Fig. S4 Sensitivity analysis examining interactions between 97 BMI single nucleotide polymorphisms and obesity lifestyle risk index on clinically measured BMI in an electronic health record biobank (*n* =17,040).** *P*_int_ value and effect estimates are for the interaction term between the 97 BMI genetic variants and the lifestyle index (continuous) on BMI in a multivariable linear regression model adjusted for age, sex, genotyping array, and 5 PCs of ancestry adding both the genetic variant and the index as covariates. The horizontal red line represents the unadjusted *P* value cut-off (*P* value =0.05). Individual interactions were considered significant at Bonferroni *P* value cut-offs accounting for the total number of interaction tests (97 tests). The top 3 interaction signals are annotated with the symbol of the nearest gene.


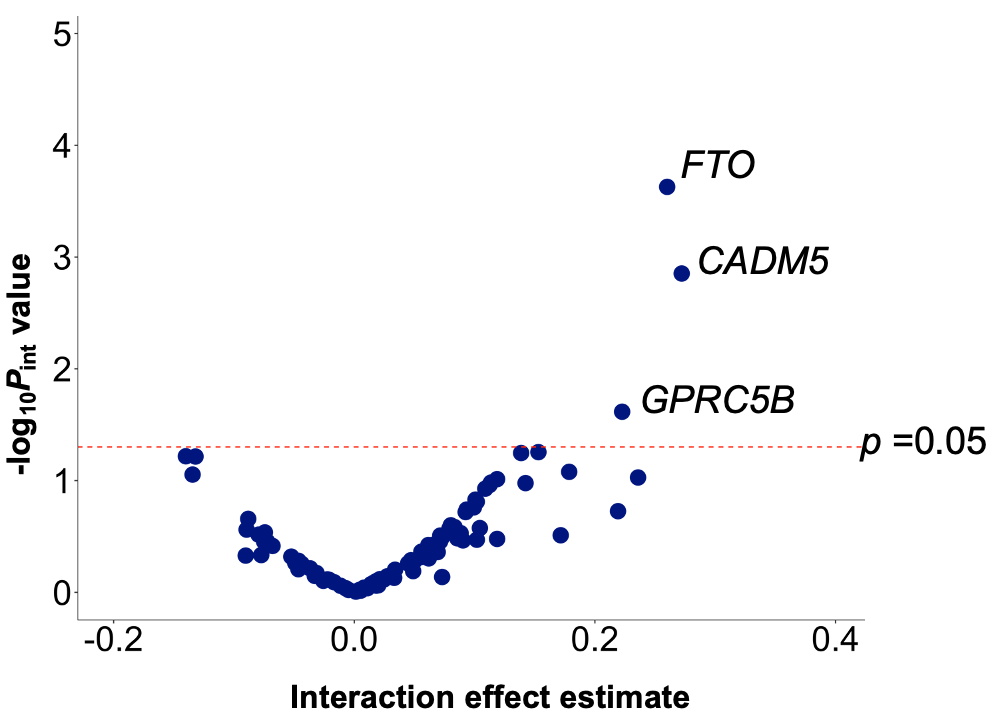


**Fig. S5 Sensitivity analysis in an electronic health record biobank (*n* =17,040) examining interactions between the obesity polygenic risk score and obesogenic lifestyle risk factors on clinically measured BMI and associations between the obesity polygenic risk score per SD and BMI stratified by lifestyle risk factor risk.** Stratified associations are shown for the lowest risk category (e.g., moderate alcohol intake (1-2 drinks per day), highest education level (Masters, doctoral or professional degree), recommended physical activity duration (≥150 minutes of moderate or high intensity exercise per week), adequate sleep duration (≥8 and <10 hours per night), never smoking, and day shift work) followed by the higher risk category (e.g., excessive or limited alcohol intake (more or less than 1 to 2 drinks per day), education level less than Masters, doctoral or professional degree, physical inactivity (<150 minutes of moderate or high intensity exercise per week), inadequate sleep duration (<8 hours or ≥10 hours per night), former smoking (associated with higher odds of obesity compared to current and never smoking; current smoking was excluded from the analysis), and night shift work) for each trait. Abbreviations: week (wk), standard deviation (SD).


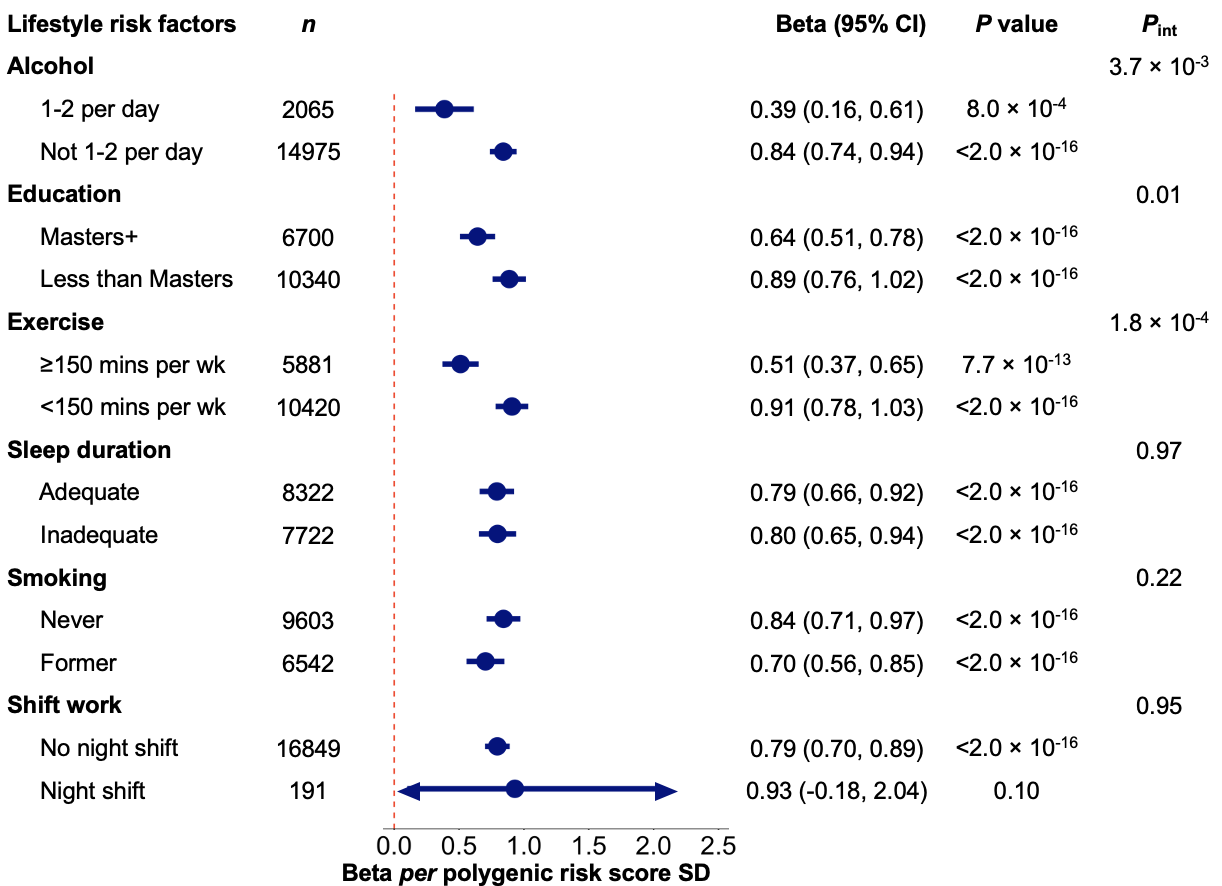

Supplement: Supplementary file 1 — Additional file 1: Figures S1-S5. Fig S1. – [Flow chart, patients analyzed in the MGB Biobank]. Fig S2. – [Associations of BMI SNPs and obesity PRS with BMI]. Fig S3. – [Associations of obesogenic lifestyle risk factors and obesity lifestyle risk index with BMI]. Fig S4. – [Interactions between 97 BMI SNPs and obesity lifestyle risk index on BMI]. Fig S5. – [Interactions between obesity PRS and obesogenic lifestyle risk factors on BMI and associations between obesity PRS per SD and BMI by lifestyle risk factor risk]. [file 12916_2021_2198_MOESM1_ESM.docx]
